# Changes in soil properties and the *phoD*-harboring bacteria of the alfalfa field in response to phosphite treatment

**DOI:** 10.3389/fmicb.2022.1013896

**Published:** 2022-11-29

**Authors:** Zhenyi Li, Jixiang Wang, Yao Wu, Jingyun Hu, Lili Cong, Chao Yang, Jinmin Fu, Juan Sun

**Affiliations:** Key Laboratory of National Forestry and Grassland Administration on Grassland Resources and Ecology in the Yellow River Delta, College of Grassland Science, Qingdao Agricultural University, Qingdao, China

**Keywords:** *Medicagosativa* (alfalfa), phosphorus fertilizer, phosphite, *phoD*-harboring bacteria, alfalfa field

## Abstract

Phosphite, a reduced form of orthophosphate, is characterized by high solubility, and transportation efficiency and can be used as potential phosphorus fertilizer, plant biostimulant and supplemental fertilizer in agriculture. However, the effects of phosphite fertilizer on soil properties and microorganisms are poorly understood. This study evaluated the effects of phosphate and phosphite fertilizers on the different forms of phosphorus, alkaline phosphatase (ALP) activity, and *phoD*-harboring bacterial community in the alfalfa (*Medicago sativa*) field. The study used four concentrations (30, 60, 90, and 120 mg P_2_O_5_ kg^−1^ soil) of phosphate (KH_2_PO_4_) and phosphite (KH_2_PO_3_) fertilizers for the alfalfa field treatment. The results showed that both phosphite and phosphate fertilizers increased the total phosphorus (TP) and available phosphorus (AP) contents in the soil. The phosphorus content of the phosphite-treated soil was lower than that of the phosphate-treated one. TP, inorganic phosphate (Pi), and AP negatively regulated ALP activity, which decreased with increasing phosphate and phosphite fertilizers concentrations. Furthermore, high-throughput sequencing analysis identified 6 phyla and 29 families, which were classified from the altered operational taxonomic units (OTUs) of the soil samples. The redundancy analysis (RDA) revealed that pH, TP, AP and Pi were significantly related to the *phoD*-harboring bacterial community constructure. The different fertilizer treatments altered the key families, contributing to soil ALP activities. Frankiaceae, Sphingomonadaceae, and Rhizobiaceae positively correlated with ALP activity in phosphite-treated soil. Moreover, the structural equation model (SEM) revealed that ALP activity was affected by the *phoD*-harboring bacterial community through altered organic phosphorus (Po), AP, total nitrogen (TN), soil organic carbon (SOC), and pH levels under phosphate fertilizer treatment. However, the effect was achieved through positive regulation of pH and AP under phosphite fertilizer. Thus, the changes in soil properties and *phoD*-harboring bacteria in response to phosphate and phosphite treatments differed in the alfalfa field. This study is the first to report the effects of phosphite on the soil properties of an alfalfa field and provides a strong basis for phosphite utilization in the future.

**Highlights:**

– Phosphite and phosphate increase the total phosphorus and available phosphorus.
– The pH was the dominant factor influencing the *phoD*-harboring bacterial community under phosphite fertilizer.
– The response of soil properties and *phoD*-harboring bacterial community to phosphate and phosphite fertilizers differed in the alfalfa field.

## Introduction

Phosphorus (P) is a macronutrient for plant growth and development, mainly absorbed by plants in orthophosphate form (Chiou and Lin, 2011; Lopez-Arredondo et al., 2014). Orthophosphate (H_2_PO_4_^2−^ or H_2_PO_4_^−^) is the only form of P utilized by microorganisms and plants in soil. However, ease of adhesion to the soil, low solubility, and poor mobility make orthophosphate a limiting essential nutrient in terrestrial and aquatic ecosystems (Lopez-Bucio et al., 2002). While 15–25% of the applied phosphate fertilizer is utilized by plants in the growing season (Lopez-Arredondo et al., 2014), excess phosphate fertilizers build up the natural phosphorus reserves in the soil and increase the availability of soluble phosphate in water bodies during leaching, leading to eutrophication and environmental pollution (Lopez-Arredondo et al., 2014; Baker et al., 2015). Plants adaptively improve their utilization of legacy soil phosphorus by changing their morphological and physiological traits and increasing the secretion of organic acids to dissolve insoluble phosphorus and acid phosphatase (ACP) to hydrolyze organic phosphorus (Po; Hinsinger et al., 2003; Wang et al., 2006; Tran et al., 2010). Additionally, a large proportion of P exists as Po in soil, which cannot be directly absorbed by plants. Alkaline phosphatase (ALP), derived from soil microorganisms, is a key enzyme involved in releasing available phosphorus (AP) from organic phosphate (Kathuria and Martiny, 2011; Turner et al., 2015; Romanyà et al., 2017). It catalyzes the hydrolysis of ester-phosphate bonds in various orthophosphate monoesters (Nannipieri et al., 2011), which is essential in plant nutrition (Nannipieri et al., 2011; Turner et al., 2015). ALP increase would reduce long-term P fertilizer inputs by increasing P cycling in the soil. Bacteria function in the biochemical cycling of P and increase ALP production in response to phosphate starvation (Nannipieri et al., 2011). The phosphate starvation regulons (Pho) have been reported in most bacteria and contain a set of co-regulated genes responsible for the synthesis and secretion of phosphatases, whose products are involved in the transportation and assimilation of inorganic phosphate (Vershinina and Znamenskaya, 2002; Apel et al., 2007).

Three homologous ALP-encoding genes, *phoA*, *phoX*, and *phoD*, have been identified in prokaryotes *via* sequence similarity and substrate specificity (Luoa et al., 2009; Kathuria and Martiny, 2011; Tan et al., 2012), whereby *phoD* gene has been shown to occur more frequently than that *phoA* and *phoX* in terrestrial ecosystem (Tan et al., 2012). Additionally, *phoD* genes have been widely identified in soil bacterial communities, and a higher correlation between *phoD* gene abundance and ALP activity has been reported (Fraser et al., 2015a; Luo et al., 2017). Therefore, *phoD* can be used as a key molecular marker for studying phosphate transformation in the soil. Since the development of the primers specific to the *phoD* gene (Sakurai et al., 2008), several studies have investigated the *phoD*-harboring bacterial communities in soil under different fertilization treatments (Tan et al., 2012; Chen et al., 2017; Luo et al., 2017; Hu et al., 2018). It has been shown that the long-term application of P fertilizer increases the diversity of the *phoD*-harboring bacteria in the grassland (Tan et al., 2012). Additionally, P depletion altered the *phoD*-harboring bacterial community and the soil pH of the grassland (Ragot et al., 2016). The response of the *phoD*-harboring bacterial community to P fertilizer is variable (Tan et al., 2012), and the inconsistency of findings suggests that *phoD*-harboring microbes are sensitive and respond differently to different fertilization modalities.

Previous studies have shown that combining inorganic and organic fertilizers increased P availability and decreased the ALP activity (Hu et al., 2018; Chen et al., 2019b; Jiang et al., 2022), and the *phoD*-harboring bacterial community was strongly regulated by the number of organic fertilization inputs in Karst soil (Hu et al., 2018). Appling cattle or swine manure increased the AP altered the *phoD* gene community and enhanced the ALP production in soil (Fraser et al., 2015b; Chen et al., 2019b). Available Po plays an important role in structuring *phoD*-harboring bacterial communities (Wei et al., 2021). Several studies have shown that long-term application of mineral P fertilizer increased the soil available P and decreased the ALP activity (Liu et al., 2020; Wang et al., 2022). Although plant morphological and physiological changes and the related soil microorganisms enhance soil P utilization, the application of P fertilizer is still the main source of soil P. Therefore, obtaining a high-efficiency phosphate resource has become important to ensure sufficient utilization of phosphate fertilizers and reduce phosphate-caused pollution.

Phosphite (Phi, H_2_PO_3_^−^), a reduced form of orthophosphate, is a phosphate analogue that can be used as potential fertilizer, plant biostimulant and supplemental fertilizer in agriculture (Achary et al., 2017; Gómez-Merino et al., 2022). The molecule is also used as a slow-release P fertilizer to provide nutrients and improve the phosphate utilization of crops (Achary et al., 2017). Phosphite is characterized by high solubility, low reactivity with soil, and high transportation efficiency (Achary et al., 2017). Phosphite can be transported by dual channels in the xylem and phloem of plants (Lovatt and Mikkelsen, 2006) and can be oxidized to orthophosphate by soil microorganisms or when exposed to air. Moreover, phosphite acts as an effective biostimulant when sufficient phosphate is provided in the growth medium (Gómez-Merino and Trejo-Téllez, 2016; Achary et al., 2017; Gómez-Merino et al., 2022) may also improve nutrient uptake and assimilation, abiotic stress tolerance and yield quality of plants (Gómez-Merino and Trejo-Téllez, 2016; Xi et al., 2020; Havlin and Schlegel, 2021).

Despite the disputable use of phosphite fertilizer due to its inability to be directly metabolized by plants (Lovatt and Mikkelsen, 2006; Thao and Yamakawa, 2009), the increasing development of biotechnology has enabled plants to convert phosphite into orthophosphate by bacterial *phosphite dehydrogenase* (*ptxD*) gene, thus making phosphite a normal P nutrient (Achary et al., 2017; Trejo-Téllez and Gómez-Merino, 2018; Havlin and Schlegel, 2021). The bacterial gene, *ptxD*, has been transformed into *Arabidopsis*, tobacco (*Nicotiana benthamiana*; Lopez-Arredondo and Herrera-Estrella, 2012), rice (*Oryza sativa*) (Manna et al., 2015), and cotton (*Gossypium hirsutum*) (Pandeya et al., 2018). It is important to explore the replacement of orthophosphate with phosphite as a phosphate fertilizer to reduce phosphate fertilizer wastage and fully use the limited P resources. This can reduce phosphate pollution, maximize the utilization of the limited P resources and help in understanding the effects of phosphite on soil and the *phoD* bacterial community. To date, studies on the effects of phosphite on soil and related microorganisms are limited.

Alfalfa (*Medicago sativa*) is an important legume forage cultivated worldwide (Guo et al., 2021); however, its yield and quality improvement are limited by phosphate availability (Fan et al., 2015). Sufficient phosphate fertilizer application has become important for obtaining high-yield and high-quality alfalfa forage (Grant et al., 2001). In China, alfalfa is mostly planted in less fertile soil, which require large amounts of phosphate fertilizer to obtain more forage with high nutritional quality. Thus, phosphite can be used as a substitute for phosphate fertilizer to maximize the utilization of the limited P resources in these soils.

Finding alternatives to phosphate fertilizers will help alleviate the shortage of P rock resources and phosphate-caused pollution and develop a sustainable agricultural development in the future. The current study aimed to evaluate the impact of phosphite fertilizers on different forms of P, ALP activity, and the relationships between *phoD*-harboring bacterial community and soil properties in the alfalfa field. We supposed that altered different forms of P and pH would be attributed to phosphite fertilizer treatment, which would also change the ALP correlated with diversity and richness of the *phoD*-harboring bacterial community. The findings provide a novel insight into soil responses to phosphite fertilizer and reference information for developing potential phosphite fertilizer.

## Materials and methods

### Experimental design

The experimental field was located at the Qingdao Agricultural University, Modern Agricultural Science and Technology Demonstration Park campus (120°04′43.29″ E, 36°26′21.51″ N), China. The average temperature, annual precipitation, and annual pressure were 13.7°C, 935.1 mm, and 100.73 kPa, respectively. Moreover, the annual sunshine duration was 2,422.3 h, with an average monthly temperature of 26°C. The chemical properties of the surface soil (0–30 cm) before the assay were 0.45 g kg^−1^ total phosphorus (TP), 27.05 mg kg^−1^ AP, 0.88 g kg^−1^ total nitrogen (TN), 77.12 mg kg^−1^ alkaline dissolved nitrogen (AN), 78.59 mg kg^−1^ available potassium (AK), and 9.84 g kg^−1^ soil organic carbon (SOC). The experimental plot area was 12 m^2^ (3 m × 4 m), and the planting density and row spacings were 2.25 g m^−2^ and 20 cm, respectively. Four different concentrations of the phosphite (KH_2_PO_3_) and phosphate (KH_2_PO_4_) used for the experiment were 30 (Phi-30 and Pi-30), 60 (Phi-60 and Pi-60), 90 (Phi-90 and Pi-90) and 120 (Phi-120 and Pi-120) mg P_2_O_5_ kg^−1^ soil. Different concentrations of potassium chloride (KCl) were added to various plots to sustain the same amount of potassium nutrient. The unfertilized field (CK) and potassium-only fertilizer treatment (CK-K) served as the controls. The treatments were applied in triplicate and all fertilizers were applied to the soil surface and tilled to a depth of 20 cm.

### Soil sampling and analysis

The soil samples were collected at a depth of 20 cm from each plot using a 5-cm diameter soil auger in June, July, and September in the first year (2020) after alfalfa harvesting. The mixed samples consisted of three random soil samples, which were divided into three subsamples after removing crop residues and large debris. One subsample from each category was placed in 50 ml centrifuge tubes and stored at −80°C for analyzing the ACP and ALP activities. One of the remaining two subsamples was dried in the shade under dark conditions for the detection of soil physicochemical characters, while the other one was stored at −80°C for DNA extraction.

The soil pH was detected with a pH meter in a 1:2.5 (v/v) mixture of soil: water suspension. A phosphomolybdate colorimetric assay was used to determine the total phosphate in the soil (Ames, 1966), while the available phosphate in the soil was measured according to a previous method (Olsen et al., 1954). Organic phosphorus (Po) and inorganic phosphorus (Pi) were determined by the high-temperature cauterization-H_2_SO_4_ leaching method, and their contents were obtained *via* the H_2_SO_4_ leaching method (for the Pi). The difference between the results obtained from these two methods was calculated for Po determination. Moreover, AK was determined by neutral ammonium acetate leaching and flame photometry, while the alkaline diffusion method was utilized determine alkaline dissolved nitrogen (AN). Total nitrogen (TN) and soil organic carbon (SOC) contents in the soil were obtained using the organic elemental analyzer (Vario EL cube, Germany).

### Determination of acid phosphatase and alkaline phosphatase activities

All soil properties were obtained after drying at 105°C. The ACP and ALP activities were determined using their respective assay kits (SACP-1-W and SAKP-1-W, Suzhou Comin Biotechnology Co, China). Briefly, 0.1 g of dry soil was incubated in toluene for 15 min and dissolved in a buffer solution. Sodium acetate–acetic acid and sodium carbonate-sodium bicarbonate buffers containing p-nitrophenyl phosphate (pNPP) were incubating at 37°C for 24 h for ACP and ALP activity detection, respectively. The reaction was stopped with aluminum sulfate hexadecahydrate, and the soil suspension was measured at 660 nm (Infinite M Plex, Tecan, Switzerland). The ACP or ALP activity was expressed as μmol pNP g^−1^ soil d^−1^, releasing 1 μmol of phenol at 37°C per day.

### DNA extraction and sequencing

The soil sample for DNA extraction was obtained during the third time cutting of alfalfa (in September). The Omega Mag-Bind soil DNA kit (Omega Inc., United States) was used to extract the microbial DNA from 0.5 g of soil samples. Subsequently, DNA concentrations were determined, and their molecular sizes were estimated by 1.2% agarose gel electrophoresis. Amplification of the *phoD* gene for high-throughput sequencing was performed as previously described by Chen et al. (2017). Briefly, primers phoD-F733 (5′-TGGGAYGATCAYGARGT-3′, containing a unique combination of different barcodes at the 5′ end) and phoD-R1083 (5′-CTGSGCSAKSACRTTCCA-3′) were used to amplify the *phoD* gene (Ragot et al., 2015). The amplification conditions were as follows: 98°C for 5 min, 5 cycles of 98°C for 30 s, 58°C for 30 s, 72°C for 45 s, and additional 28 cycles of 98°C for 30 s, 55°C for 30 s, 72°C for 45 s, 72°C for 5 min. The amplicons were purified, quantified on a Microplate reader (BioTek, FLx800), and pooled together for normalization in an equimolar mixture. Thereafter, mate-pair sequencing of the 2 × 300 base pair (bp) sequences was conducted on the Miseq system using MiSeq Reagent Kit v3 (600-cycles-PE) (Illumina, MS-102-3,003) for library generation at Shanghai Personalbio Technology Co., Ltd. (Shanghai, China).

### Processing of illumina Miseq reads

Metagenomics was performed using QIIME 2 software version 2019.4 (Bolyen et al., 2019), while the operational taxonomic unit (OTU) clustering was conducted using the Vsearch (v2.13.4) software (Rognes et al., 2016). Briefly, raw sequence data were demultiplexed using the demux plugin, followed by paired-end primer removal using a cutadapt plugin (Martin, 2011). Sequences were then merged, filtered, and dereplicated using fastq merge pairs, fastq filter, and derep full-length functions of the Vsearch software. Unique sequences were then clustered at a 98% identity threshold *via* cluster size, followed by chimeric sequences removal using the uchime denovo. The non-chimeric sequences were re-clustered at a 97% identity threshold to generate OTU tables and representative sequences. The representative sequences were aligned using mafft (Katoh et al., 2002) and then used to construct a phylogenetic tree *via* the fasttree2 (Price et al., 2010). Thereafter, the singleton OTUs and their representative sequences were eliminated from the OTU tables. The sequences were then aligned using the BROCC algorithm (Nilsson et al., 2006) of the non-redundant (NR) database and annotated based on the recommended parameters. The qiime feature-table rarefy function of QIIME 2 was used to level the OTU table to a depth of 95% of the minimum sequence size.

### Statistical analyses

Prior to the statistical analyses, the normality and homogeneity of the data were investigated according to the Shapiro–Wilk test (Chadha et al., 2019). If the data did not conform to the normal distribution, the arcsine-transformed or log-transformed tests would then be used for the statistical analysis. Pearson correlation and analysis of variance (ANOVA) were performed on the soil properties and *phoD*-harboring bacterial diversity using SPSS software (version 26.0, IBM SPSS Inc., Chicago, IL, United States), with P fertilizer type and concentration as independent variables. Moreover, Spearman correlation coefficients were used to determine the potential correlations between the soil properties and *phoD-*harboring bacteria phylum, family, and genus. Principal coordinate analysis (PCoA) was used to evaluate the similarity or dissimilarity of the *phoD*-harboring bacterial community by calculating the Bray-Crutis distance of various samples. The composition dissimilarity of the *phoD*-harboring bacterial community was assessed by analysis of similarities (Anosim) in the vegan R package (version 4.1.1).^1^ For direct gradient analysis, the detrended correspondence analysis (DCA) was used to determine whether canonical correspondence analysis (CCA) or redundancy analysis (RDA) fitted our data. Moreover, the vegan package was used for RDA to determine the effects of soil properties on the structure of the *phoD*-harboring bacterial community. Duncan tests were used to determine the significant difference (*p* < 0.05) among the soil properties of treatments.

A structural equation model (SEM) was established using the AMOS software (IBM SPSSS 26.0.0; Malaeb et al., 2000) to illustrate the relationship among soil properties, *phoD*-harboring bacterial diversity and richness, and ALP activity under the different P fertilizer. The Chao1 index was used to describe the microbial community richness, and the Shannon index was used to represent the microbial community diversity (Wang et al., 2022; Xu et al., 2022). Model accuracy was tested with goodness-of-fit index (CFI) (*p* > 0.05) and Akaike information criterion (AIC; Markland, 2007).

## Results

### Soil properties of the alfalfa field

**Figure 1:**
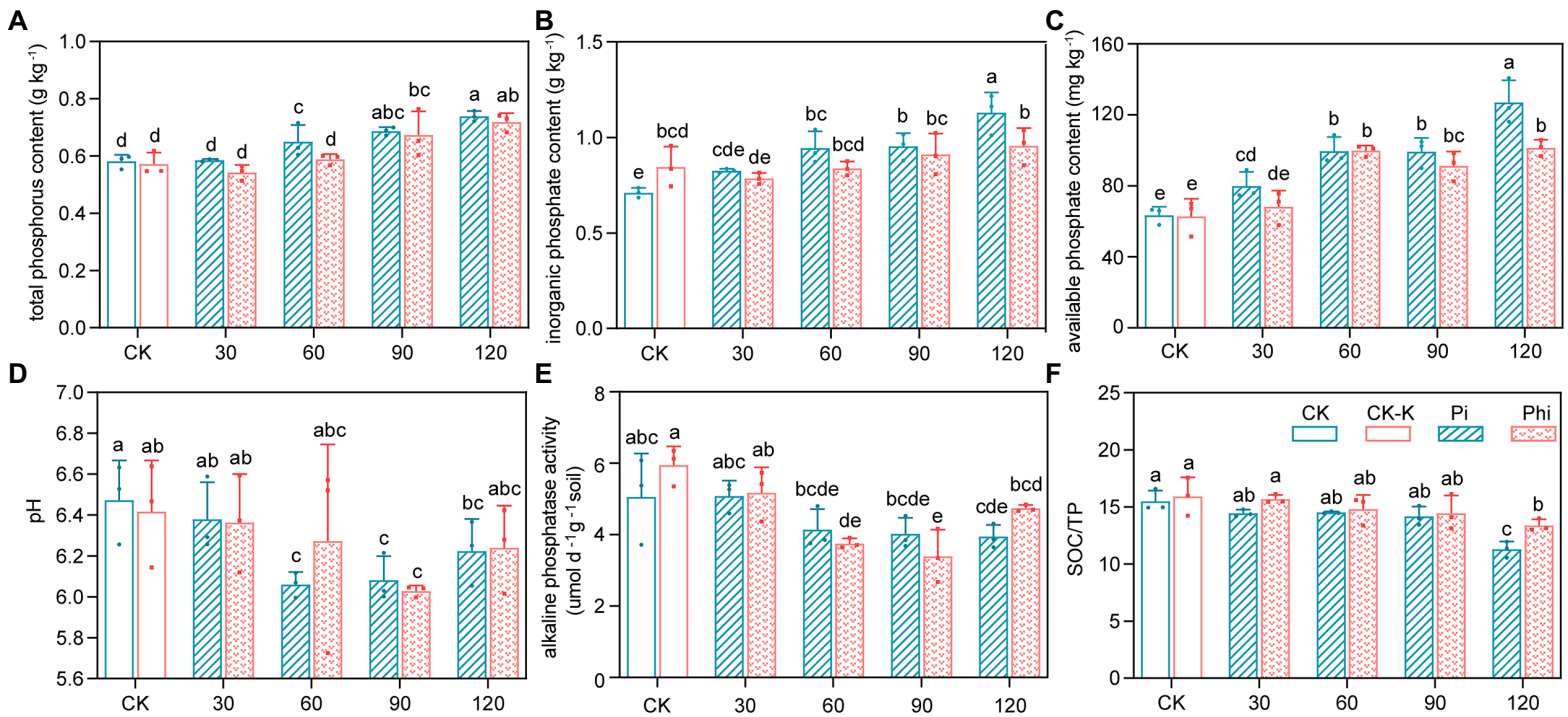
Soil properties of the phosphate- and phosphite-fertilized fields. **(A)** Total phosphorus content. **(B)** Inorganic phosphorus content. **(C)** Available phosphorus content. **(D)** pH value. **(E)** Alkaline phosphatase activity. **(F)** Soil organic carbon to total phosphorus ratio. The treatment levels are presented as the average value ± standard error (*n* = 3). Different lowercase letters indicate significant differences (*p* < 0.05). CK, non-fertilized control; CK-K, potassium-fertilized control; Phi, phosphite fertilizer treatment; Pi, phosphate-fertilized treatment. The values 30, 60, 90, and 120 represent the treatment concentrations.

The different concentrations of various P fertilizer treatments significantly (*p* < 0.05) affected the TP, Pi, and AP contents and the pH value (Figure 1; Supplementary Tables S1, S2). The contents of TP, Pi, and AP increased with fertilizer concentration for both phosphate and phosphite treatments, with the peak appearing at 120 mg kg^−1^ (Figure 1). The 30, 60, 90, 120 mg kg^−1^ of phosphate fertilizer increased the TP content by 2.72, 7.39, 12.76, and 34.44%, while same concentrations of the phosphite fertilizer increased the content by −3.69%, 1,12, 4.66, and 18.60%, respectively (Figure 1A). The AP content ranged from 51.51 to 165.89 mg kg^−1^, and was the lowest in unfertilized field (CK). The AP increased by 25.91, 56.84, 56.07, and 99.83% under phosphate treatment and by 7.42, 57.14, 43.78 and 59.76% under phosphite treatment at 30, 60, 90, and 120 mg kg^−1^ level of fertilizer, respectively (Figure 1C). Moreover, the contents of TP, Pi, and AP in phosphite-treated soils were lower than those treated with phosphate. Soil TN and SOC contents were significantly higher at Pi-90 and Phi-120 but lower at Pi-30 (*p* < 0.05; Supplementary Table S2). Moreover, the pH initially decreased with increasing concentration at 30 to 90 mg kg^−1^ level of fertilizer (*p* < 0.05; Figure 1D). Soil ALP activity ranged from 3.34 to 6.59 μmol pNP g^−1^ soil d^−1^. Furthermore, ALP activity decreased by 18.19, 20.51, and 22.09% under phosphate and by 25.83, 32.87, and 6.32% under phosphite fertilizer treatments at 60, 90 and 120 mg kg^−1^ dose, with the highest ALP activity being under CK and CK-K treatments (*p* < 0.05; Figure 1E). SOC/TP only decreased under 120 mg kg^−1^ fertilizer treatment (Figure 1F).

**Figure 2:**
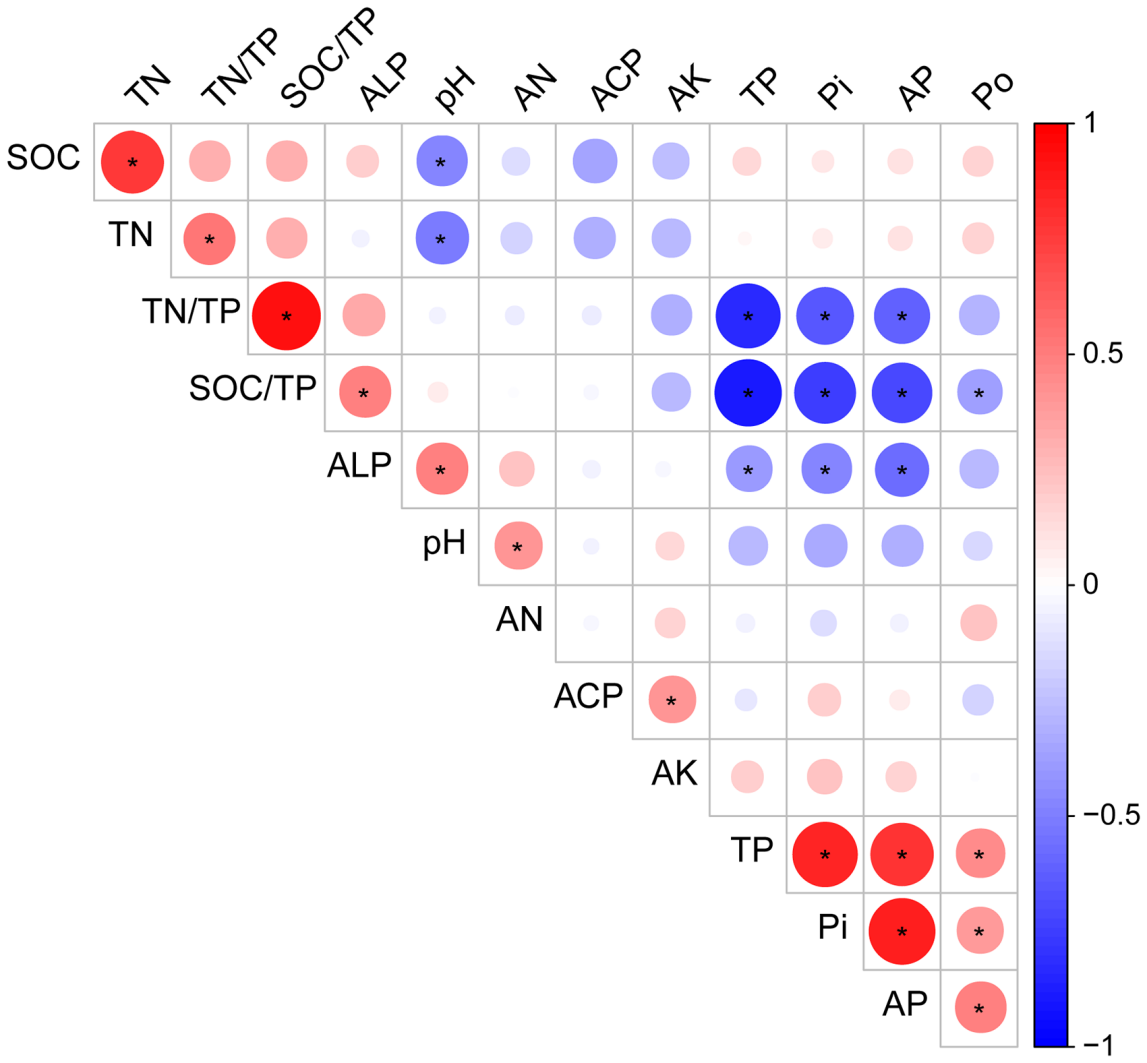
Correlation analysis of the soil properties of the alfalfa field under phosphorus fertilizer treatment. The red circle represents a positive correlation, while the blue circle represents a negative correlation. TP, total phosphorus; Po, organic phosphorus; Pi, inorganic phosphorus; AP, available phosphorus; TN, total nitrogen; AN, alkaline dissolved nitrogen; AK, available potassium; SOC, soil organic carbon; ALP, alkaline phosphatase activity; ACP, acid phosphatase activity; TN/TP, total nitrogen to total phosphorus ratio; SOC/TP, soil organic carbon to total phosphorus ratio. The color intensities indicate correlation coefficients between −1.0 and 1.0. The asterisk “*” represents significant correlation at significance level of *p* < 0.05.

We used the Pearson correlation to determine the association between the different soil properties, and the results showed that TP, Po, Pi, and AP were positively correlated (*p* < 0.05; Figure 2). ALP activity was negatively correlated with TP, Pi, and AP, but positively correlated with pH and SOC/TP (*p* < 0.05). However, the pH value was negatively correlated with SOC, TN, and AN (*p* < 0.05; Figure 2).

### Miseq-sequencing data and *phoD*-harboring bacterial diversity

**Figure 3:**
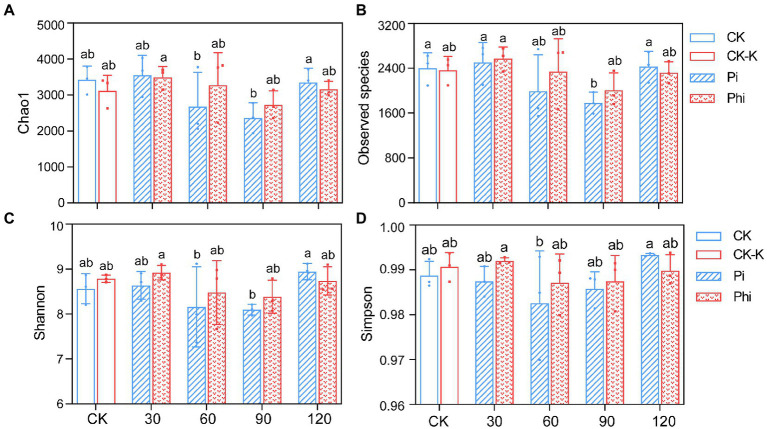
The α-diversity of the *phoD*-harboring bacterial community. **(A)** Chao1 index. **(B)** Observed species index. **(C)** Shannon index. **(D)** Simpson index. The treatment levels are represented as the average value ± standard error (*n* = 3), and the different letters indicate the significant differences (*p* < 0.05). CK denotes non-fertilized control; CK-K, potassium-fertilized control; Phi represents phosphite fertilizer; Pi, phosphate fertilizer. The values 30, 60, 90, and 120 in *X*-axis indicate 30, 60, 90, and 120 mg P_2_O_5_ kg^−1^ soil, respectively.

A total of 1,367,214 high-quality sequences containing 1,568 to 2,792 OTUs were obtained from various soil samples, with an average of 45,574 sequences per sample. We rarified the number of *phoD* gene OTUs to 3,370 for each sample to ensure richness and community structure comparability of the *phoD*-harboring bacteria among different samples (Supplementary Table S3). The parallelizing rarefaction curve indicated that the sequencing data sufficiently reflected the microorganism diversity (Supplementary Figure S1). The α-diversity exhibited Chao1, Observed species, and Shannon indices decreased with the increase of fertilizer concentration from 30 to 90 mg kg^−1^ (Figure 3). Plots treated with phosphate fertilizer had lower values than those with phosphite fertilizer at different concentrations, except for the highest concentration (Figure 3).

**Figure 4:**
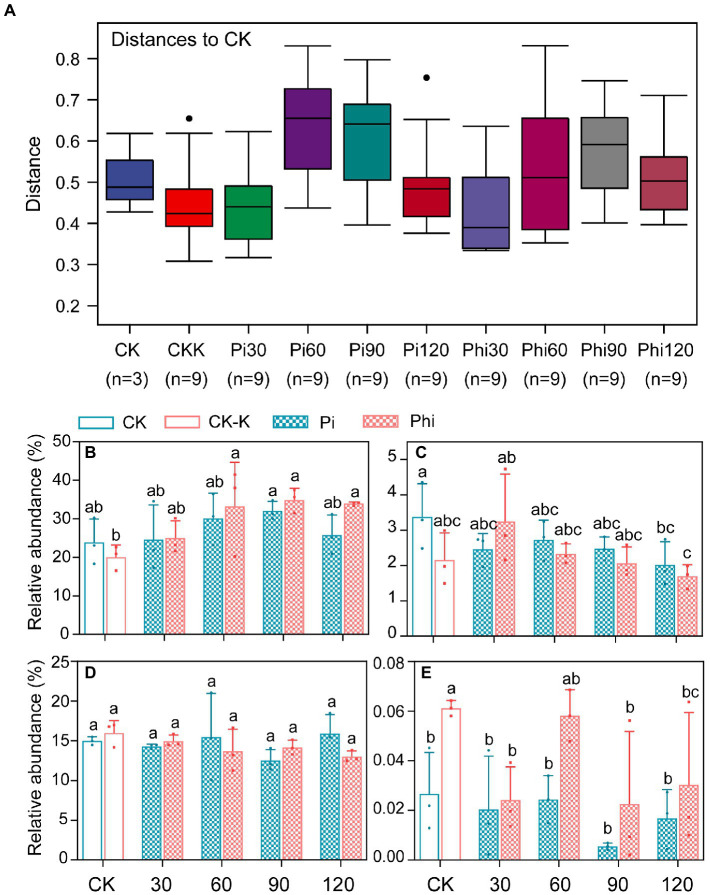
The differences between treated groups and control group **(A)** and the relative abundance of phyla contained Proteobacteria **(B)**, Firmicutes **(C)**, Actinobacteria **(D),** and Acidobacteria **(E)** under phosphate and phosphite fertilizers. **(A)** The box represents the distance between samples internal group and the distance between control sample and other groups. The point outside the upper and lower edges represents an abnormal value. Phi, phosphite fertilizer; Pi represents phosphate fertilizer. The values 30, 60, 90, and 120 mg P_2_O_5_ kg^−1^ soil indicate the fertilizer concentration used in the study. The levels of columns are represented as the average value ± standard error (*n* = 3), and the different letters indicate the significant differences (*p* < 0.05).

PCoA and Anosim were employed to analyze β-diversity. It was distinguished that the OTUs of groups affected by different concentrations of phosphate and phosphite fertilizer (Supplementary Figure S2). The distance was also exhibited between CK and treated groups using Anosim analysis (Figure 4A). All the *phoD* sequences were classified into 6 phyla, 10 classes, 23 orders, 29 families, 10 genera, and 3 species (Supplementary Table S4). The phyla contained Proteobacteria, Actinobacteria, Planctomycetes, Firmicutes, Acidobacteria, and Cyanobacteria (Supplementary Figure 3A). Among these bacteria, Proteobacteria, Actinobacteria, and Planctomycetes accounted for 50.27% of the OTUs (Figure 4; Supplementary Figures S3B,C). Proteobacteria constituted 28.08% of all sample OTUs, which was significantly affected by the different concentrations of the phosphate and phosphite fertilizers (*p* < 0.05; Supplementary Figure S3A). Firmicutes and Acidobacteria constituted 2.55% of the sequences.

### Environmental factors associated with *phoD*-harboring bacterial community

**Figure 5:**
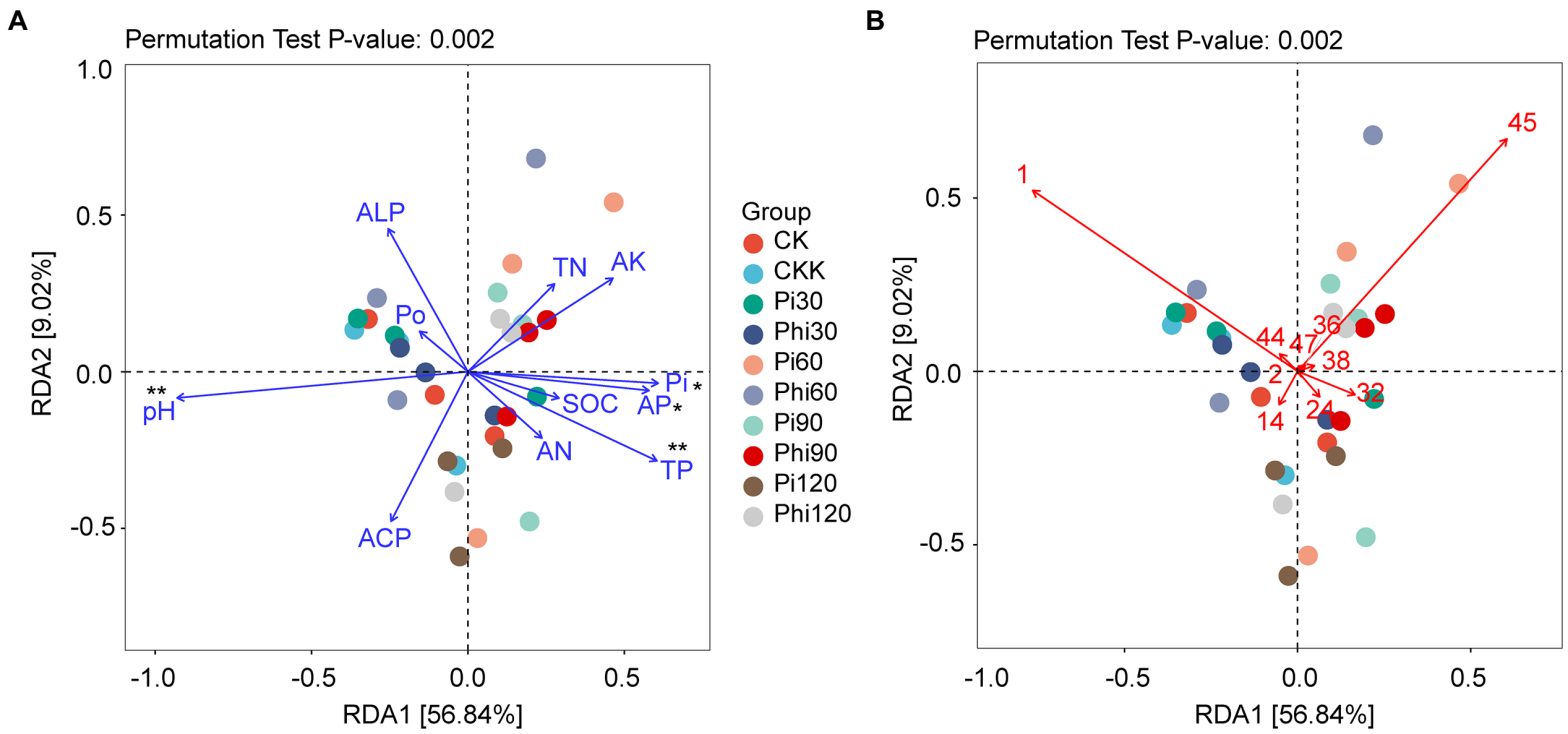
The redundancy analysis (RDA) plot showing the relationships between environmental factors and the OTUs profiles of the *phoD*-harboring bacterial community. **(A)** Significant properties exhibited in RDA. **(B)** Significant family microorganisms exhibited in RDA. The blue lines represent the environmental variables, and the colored dots denote soil samples from the different fertilizer treatments. See Figure 2 for the defined abbreviations. (**B)** The number represents family name, 1, unassigned family; 2, Vicinamibacteraceae; 14, unclassified family; 24, unclassified family; 32, Acetobacteraceae; 36, unclassified family; 38, Comamonadaceae; 44, Pseudomonadaceae; 45, Xanthomonadaceae; 47, unclassified family. Phi represents phosphite fertilizer; Pi denotes phosphate fertilizer. The values 30, 60, 90, and 120 represent the treatment concentrations. The asterisk “*” represents significant relationships (*p* < 0.05), while the double asterisk “**” represents highly significant relationships (*p* < 0.01).

Since DCA showed that the axis length of the gradient length was from 0.37 to 0.74 (Supplementary Table S5), which was less than 3.0, we determined that RDA was more reliable than CCA. RDA1 and RDA2 explained 56.84 and 9.02% of the total variance, respectively, and jointly explained 65.86% of *phoD*-harboring bacterial community variance (Figure 5A). The RDA showed a significant correlation of TP, Pi, AP, and pH with the *phoD*-harboring bacterial community structure (Figure 5A). Moreover, RDA1 positively correlated with AP but negatively with pH. Some important families dominated bacterial structure were also found through RDA. As shown in Figure 5B, Xanthomonadaceae, Acetobacteraceae and Pseudomonadaceae were more abundant in the *phoD*-harboring bacterial community. Xanthomonadaceae was negatively correlated with Acetobacteraceae (Figure 5B). Similarly, Pseudomonadaceae negatively correlated with Acetobacteraceae (Figure 5B).

**Figure 6:**
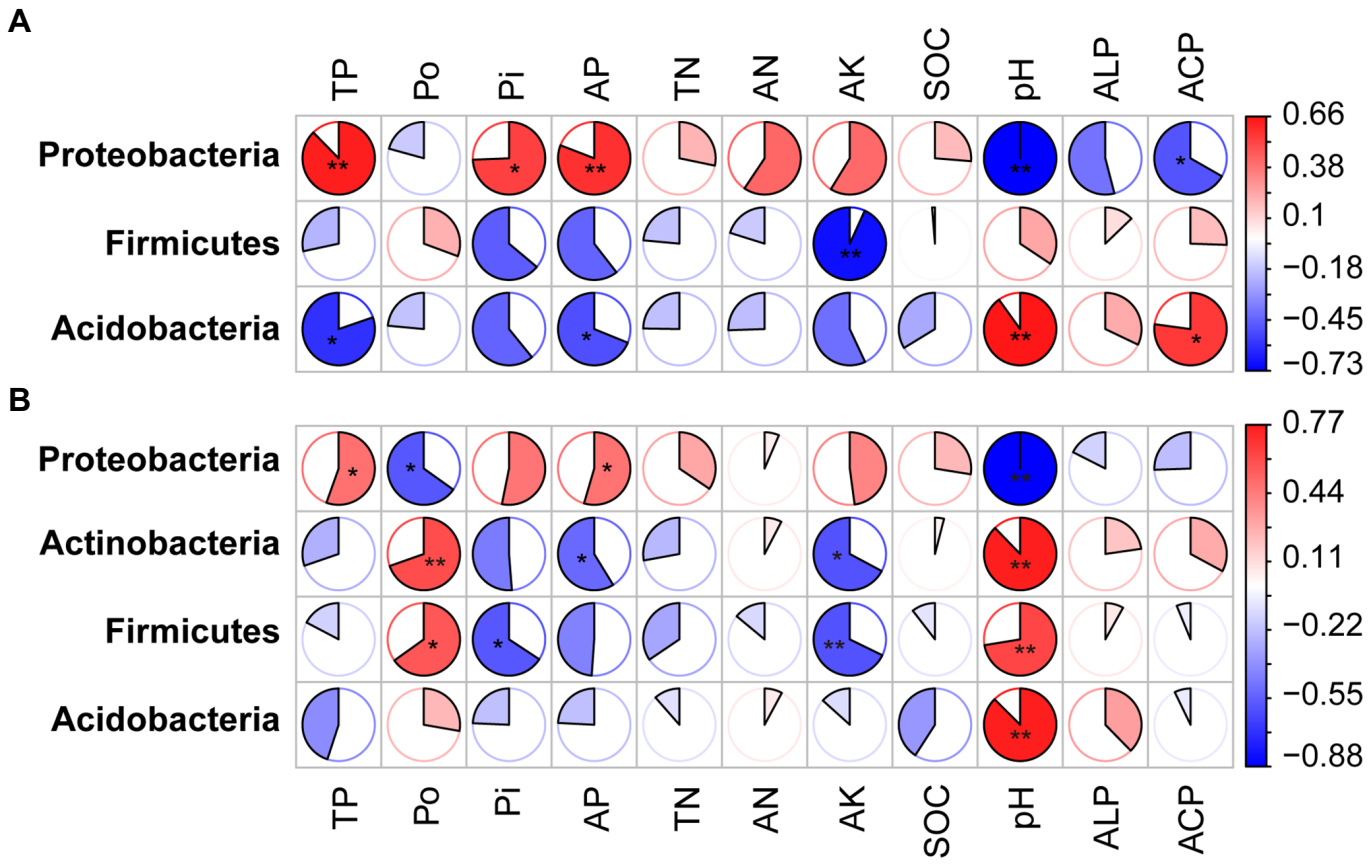
The matrix of the Spearman correlation coefficients showing the phyla of *phoD*-harboring bacteria and soil physicochemical properties under phosphate fertilizer **(A)** and phosphite fertilizer **(B)**. See Figure 2 for the defined abbreviations. The circle indicates larger correlation coefficients, and only significant correlations (*p* < 0.05) are included. The asterisk “*” represents significant relationships (*p* < 0.05), while the double asterisk “**” represents highly significant relationships (*p* < 0.01). The coefficient Spearman correlation ranges from −0.73 (blue) to 0.66 (red) under phosphate fertilizer and − 0.88 (blue) to 0.77 (red) under phosphite fertilizer.

**Figure 7:**
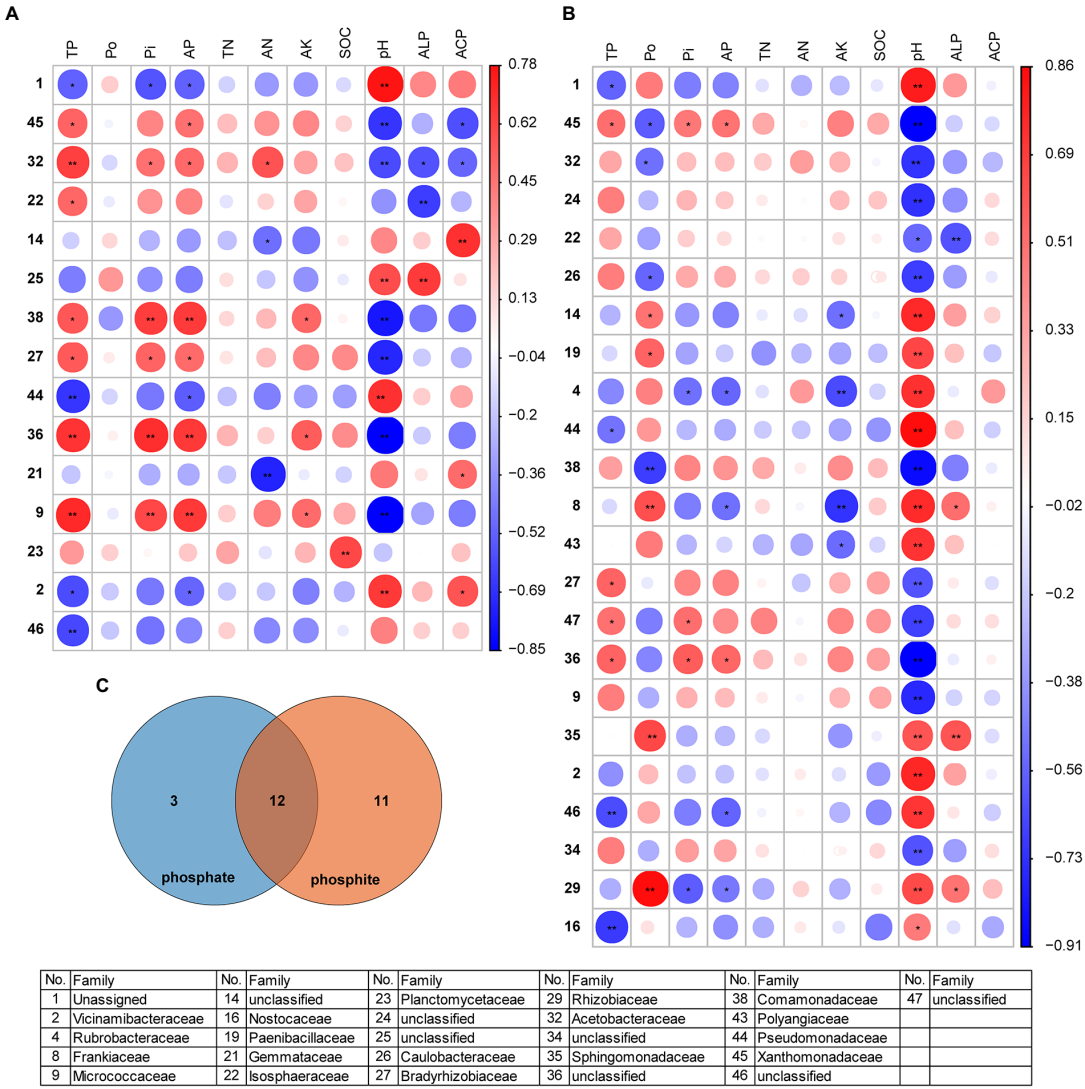
The matrix of the Spearman correlation coefficients showing the *phoD*-harboring bacterial families and soil physicochemical properties under phosphate fertilizer **(A)** and phosphite fertilizer **(B)**. Venn diagram of the families under phosphate- and phosphite-treated field **(C)**. The circle indicates larger correlation coefficients, and only significant correlations (*p* < 0.05) are included. See Figure 2 for the defined abbreviations. The asterisk “*” represents significant relationships (*p* < 0.05), while “**” represents highly significant relationships (*p* < 0.01). The coefficient Spearman correlation ranges from −0.85 (blue) to 0.78 (red) under phosphate fertilizer and − 0.91 (blue) to 0.86 (red) under phosphite fertilizer.

Spearman correlation was performed to identify the relationships between phyla, families and genera, and the soil physicochemical indicators. Under phosphate fertilizer treatment, TP, Pi, AP, AN, and pH influenced the *phoD*-harboring bacterial community. The TP, Po, Pi, AP, pH, and ALP activity correlated with the *phoD*-harboring bacterial community under phosphite fertilizer (Figures 6, 7 and Supplementary Figure S5). We also found that the relative abundance of Proteobacteria positively correlated with TP, Pi, AP, and TN contents but a negative relationship with the pH and ALP activity (Figure 6). The Acidobacteria negatively correlated with the TP, Pi, AP, TN, and AK under phosphate fertilizer, while positively correlated with pH and ACP under phosphate fertilizer (Figure 6A). Proteobacteria, Actinobacteria, Firmicutes and Acidobacteria correlated with pH under phosphite fertilizer (Figure 6B). The relative abundance of Proteobacteria gradually increased with the fertilizer concentration and reached the highest level at 90 mg kg^−1^, significantly higher than that of CK (*p* < 0.05; Figure 4B). Conversely, the relative abundances of Firmicutes and Acidobacteria gradually decreased with the increase in fertilizer concentration and reached the lowest level at 90 mg kg^−1^, significantly lower than that of CK (*p* < 0.05, Figure 4).

Some common and specific families, such as Acetobacteraceae, Xanthomonadaceae, Pseudomonadaceae, and others were identified under phosphate and phosphite fertilizers (Figure 7; Supplementary Table S6). However, Rubrobacteraceae, Frankiaceae, and others only appeared in phosphite-treated soil. Frankiaceae, Sphingomonadaceae, and Rhizobiaceae positively correlated with ALP activity in phosphite-treated soil (Figure 7B). The relative abundance of families differed under different concentrations of phosphate and phosphite fertilizers, such as Acetobacteraceae, Comamonadaceae, Pseudomonadaceae, Frankiaceae, and others (Supplementary Figure S4). Under phosphite fertilizer, the genera were also significantly correlated with pH (Supplementary Figure S5). However, just *Paludisphaera* and *Bradyrhizobium* in both two treatments, *Sinorhizobium* in phosphate and *Bacillus* in phosphite were correlated with the changed soil properties (Supplementary Figure S5). Thus, these results indicated that pH maybe plays a very important role in the *phoD*-harboring bacterial community under phosphite treatment.

### The response difference of soil to phosphite with phosphate fertilizers

**Figure 8:**
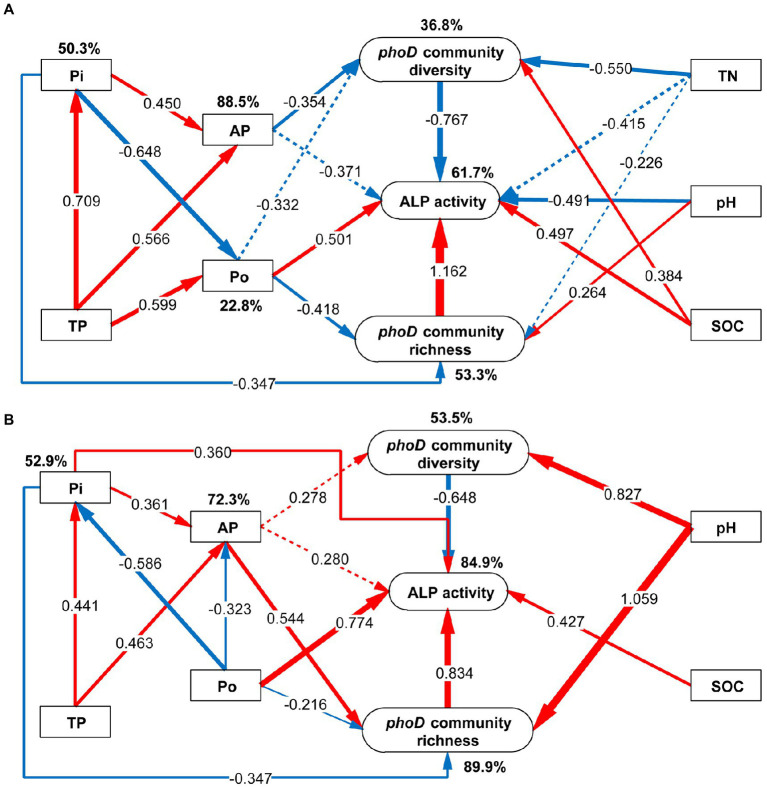
Structural equation model (SEM) of the diversity and richness of *phoD*-harboring bacterial community, alkaline phosphatase (ALP), and soil properties under different phosphorus fertilizer treatments. **(A)** Under phosphate fertilizer treatment, the SEM parameters, including χ2 = 27.92, df = 18, χ2/df = 1.551, *p* = 0.063, GFI = 0.939, and RMSEA = 0.175. **(B)** Under phosphite fertilizer treatment, the SEM parameters, including χ2 = 14.37, df = 12, χ2/df = 1.198, *p* = 0.278, GFI = 0.994, and RMSEA = 0.105. See Figure 2 for the defined abbreviations. The *phoD* community diversity and richness were described by Chao1 and Shannon indexes, respectively. The red and blue arrows represent positive and negative effects, respectively. The thickness of the line represents the degree of influence, and the dotted line represents insignificant influence. The value on the line represents standardized path coefficients.

To understand the interaction between microorganisms and soil properties, we utilized SEM to determine the direction and intensity of their interactions under phosphate and phosphite fertilizers. The SEM explained 61.7 and 84.9% of variations in ALP activity under phosphate and phosphite treatment, respectively (Figure 8). The common exhibitions between phosphate and phosphite fertilizer were; (1) TP increased the supply of Pi and AP since AP was transformed from Pi and TP; (2) Po and Pi had a negative standardized path coefficient, which negatively influenced transformation between them in soil; (3) ALP activity was promoted by Po, SOC, and *phoD*-harboring bacterial richness, but inhibited by *phoD*-harboring bacterial diversity; (4) ALP activity was not affected by TP and AP but was affected by the richness and diversity of the *phoD*-harboring bacterial community (Figure 8).

Furthermore, there were some differences between phosphate and phosphite fertilizer stimulations; for example, the SEM results showed a variance of 61.7% in ALP activity under phosphate fertilizer, while 84.9% variance was exhibited under phosphite fertilizer (Figure 8). ALP activity was directly affected by pH (path coefficient = −0.491), SOC (path coefficient = 0.497), Po (path coefficient = 0.501), and *phoD*-harboring bacterial community richness (path coefficient = 1.162) and indirectly effected by TP, AP, Pi, and TN when exposed phosphate fertilizer. However, ALP activity was directly affected by SOC (path coefficient = 0.427), Po (path coefficient = 0.774), Pi (path coefficient = 0.360), and *phoD*-harboring bacterial community richness and diversity and indirectly effected by AP, Po and pH. The diversity of the *phoD*-harboring bacterial community was reduced by TN and AP under phosphate fertilizer and not under phosphite fertilizer. Meanwhile, TN did not affect the *phoD* community diversity and ALP activity under phosphite fertilizer. The *phoD* gene community diversity was significantly affected by SOC under phosphate fertilizer but not under phosphite fertilizer. Moreover, the pH positively influenced the diversity and richness of *phoD* community as shown by the standardized path coefficients of 0.827 and 1.059 under phosphite fertilizer, compared to the phosphate fertilizer (Figure 8). Thus, we speculated that *phoD* community structure mainly affected by pH under phosphite fertilizer treatment. Under phosphate fertilizer, increased AP inhibits ALP activity by inhibiting *phoD*-harboring bacterial community diversity.

## Discussion

The limiting P resources and the increasing phosphate-caused pollution necessitate reducing the P fertilizer inputs and developing phosphate-substituting fertilizer in the future. Phosphite is widely used as a fertilizer, biostimulant, and potential herbicide (Lopez-Arredondo and Herrera-Estrella, 2012; Manna et al., 2015; Achary et al., 2017; Pandeya et al., 2018; Trejo-Téllez and Gómez-Merino, 2018; Havlin and Schlegel, 2021; Gómez-Merino et al., 2022). However, there are a few reports on how phosphite influences soil and related microorganisms. This study compared the changes in soil properties and *phoD*-harboring bacterial community in the alfalfa field in response to phosphite and phosphate fertilizers.

### Soil properties were altered by phosphate and phosphite fertilizers

Appling inorganic phosphorus or organic phosphorus fertilizers effectively improves phosphate availability in soil (Garg and Bahl, 2008; Keller et al., 2012; Fraser et al., 2015b; Luo et al., 2017). In this study, the soil TP and AP contents significantly increased in both phosphate and phosphite treatments. It is no surprise that P fertilizer increased TP and AP. However, increased AP could have stemmed from easily soluble phosphite in phosphite-treated soil, instead of available phosphate. However, the phosphite-induced increase in TP and AP was lower than that of phosphate, possibly because phosphite has high solubility and is difficult to be fixed soil (Achary et al., 2017), making it easily acquired by plants and left in soil. Phosphite also provides nutrients to plants as a slow-release fertilizer when oxidized to phosphate by microorganisms (Ohtake et al., 1996). The different concentrations of phosphate and phosphite fertilizers did not significantly affect the contents of Po, AN, and AK and ACP activity. In the present study, we concluded that the altering changes in soil properties caused by phosphite and phosphate fertilizers were the same.

It has been reported that inorganic fertilizers decrease soil ALP activity due to reduced soil pH (Luo et al., 2017), consistent with our results. We observed that the soil pH and ALP activity decreased significantly (*p* < 0.05) with the increasing fertilizer concentration. The pH value and ALP activity of the phosphite-treated soils decreased more rapidly than that of the phosphate-treated soils. This is because phosphite is readily fixed in the soil, transformed by microorganisms, and absorbed by plants. Also, the low AP content in the phosphite-treated soil weakened the inhibition of ALP activity. There was a negative correlation between AP content and ALP activity in soils (Fraser et al., 2015a) since the ALP activity decreased significantly with increasing AP in soil. Since ALP activity is regulated by Pi, low phosphate levels induce the expression of alkaline phosphatase for microbial growth (Saha et al., 2008; Zhang et al., 2014). However, AP is affected by both chemical and biological factors. Thus, the association between ALP activity and AP may differ across studies due to varying external factors (Hu et al., 2018).

### Changes in the *phoD*-harboring bacterial community

Long-term fertilization also exerts severe and lasting effects on soil microbial community structure and function (Liang et al., 2015). The diversity of the *phoD*-harboring bacterial community was also affected by organic and inorganic fertilizer treatments (Tan et al., 2012; Chen et al., 2017; Luo et al., 2017). Since *phoD*-harboring bacteria play important roles in the uptake of phosphate or other nutrients (Vershinina and Znamenskaya, 2002). The *phoD*-harboring bacteria diversity, abundance and community richness was decreased under long-term phosphate-deficient soils (Zhong and Cai, 2007; Tan et al., 2012; Chen et al., 2017; Farmer et al., 2018; Wang et al., 2022), was consistent with our results. This is because that a large amount of P input suppress the growth and reproduction of *phoD*-harboring bacteria (Wei et al., 2019). Although *phoD*-harboring bacteria are taxonomically diverse, only a few OTUs are induced for ALP synthesis (Fraser et al., 2015b). In the present study, the ALP activity was higher in control, while soils treated with 30 mg kg^−1^ of fertilizer had lower AP, suggesting that *phoD*-harboring bacteria synthesizing ALP are preferentially selected under phosphate deficiency conditions with limited soil bacterial diversity. In the present study, the diversity of the *phoD*-harboring bacteria community was higher in phosphite-treated soil than that in the phosphate-treated soil, indicating that the *phoD*-harboring bacterial community in phosphite-treated soils had higher abundance and homogeneity. This reason may be because phosphite is easily absorbed by plants, reducing its concentration in the soil, which the *phoD*-harboring diversity was reduced by phosphate more than that by phosphite. ALP activity and bacterial community structure are strongly correlated with soil pH (Ragot et al., 2016).The reduced soil pH due to decreased TP concentration in the soils, affected the *phoD*-harboring bacteria diversity (Fierer and Jackson, 2006). This might be because phosphate and phosphite treatments reduced soil pH, lowering *phoD*-harboring bacteria diversity. Short-term fertilizer treatment caused varying effects on the *phoD*-harboring bacteria community compared with long-term treatment. Therefore, the effect of long-term phosphite fertilizer on soil *phoD*-harboring bacterial community should be further explored.

### Relationship between environmental factors and the *phoD*-harboring bacterial community

SOC, TN, and AP play dominant roles in shaping the *phoD*-harboring bacterial community (Wang et al., 2022). Notably, enzyme production needs more energy and nutrients, which requires large amounts of carbon and nitrogen. The pH and phosphate depletion affected the *phoD*-harboring community structure in the soil (Ragot et al., 2016). Long-term application of nitrogen fertilizer increased ALP activity and abundance of *phoD*-harboring bacteria in wheat-maize rotation soil (Li et al., 2014). The analysis also showed that soil phosphorus, SOC, and pH affected the *phoD*-harboring bacterial community (Ragot et al., 2016; Luo et al., 2017; Wang et al., 2022). pH is the main driving factor, accounting for 42.8% of the change of *phoD* community composition (Zhang et al., 2020). In this study, soil pH also had a greater effect on the *phoD*-harboring bacterial community than others soil indicators. In summary, the contents of TP, Pi, and AP and the *phoD*-harboring bacterial community affected the ALP activity in soil.

Under long-term P fertilization or phosphate depletion, the dominant *phoD*-harboring phyla Proteobacteria, Actinobacteria, Cyanobacteria, Firmicutes, and Planctomycetes were identified (Tan et al., 2012; Ragot et al., 2016; Luo et al., 2017; Liu et al., 2020; Jiang et al., 2022; Wang et al., 2022). The Proteobacteria and Actinobacteria are the most abundant *phoD*-harboring bacteria in soils (Zhang et al., 2020). The *Bradyrhizobium*, *Paludisphaera,* and *Phenylobacterium* were the dominant *phoD*-harboring bacteria at the genus level (Lagos et al., 2016; Luo et al., 2017). Our results showed that the dominant *phoD*-harboring bacteria, Proteobacteria, Firmicutes, and Acidobacteria, were similar between phosphate- and phosphite-treated soils, consistent with the previous studies (Tan et al., 2012; Ragot et al., 2016). In the identified families, Frankiaceae, Sphingomonadaceae, and Rhizobiaceae positively correlated with ALP activity, just like in phosphite-treated soil, with potentially important roles in transformation of AP. The *phoD* gene abundance were reduced in grassland soil was xposured to short-term phosphate fertilizer application (Ikoyi et al., 2018).

The matrix of Spearman’s correlation coefficients showed that all *phoD*-harboring bacteria significantly correlated with the environmental factors, but not each *phoD*-harboring bacteria was associated with a single environmental factor. The *phoD*-harboring bacteria Proteobacteria negatively correlated with the ALP activity (Chen et al., 2019a). Pseudomonas promotes plant growth and development (Miller et al., 2010) and enhances the mineralization of Po (Giles et al., 2014; Alori et al., 2017). It was reported that nitrate and ammonia nitrogen were the main drivers of the *phoD*-harboring bacterial community in the river mouth zone (Zhang et al., 2020). In this study, TP, Pi, AP, TN, and AN increased with the relative abundance of *phoD*-harboring bacteria *Bradyrhizobium*, important for the N-P interactive regulation in soil. *Proteobacteria* (e.g., *Rhizobaiales*) acts as a decomposer in the carbon cycle (Stursova et al., 2012) and positively correlates with SOC in copiotrophic environments (Bastida et al., 2015). Conversely, Actinobacteria negatively correlated with SOC in oligotrophic environments (Fierer et al., 2012). In the present study, Proteobacteria was the dominant bacteria, which played an important role in soil carbon metabolism (Hayatsu et al., 2008). Actinobacteria (e.g., *Actinomycetales*) and Cyanobacteria (e.g., *Gloeobacterales*) were also involved in N2 fixation and the N cycle, similar to nitrobacteria (Hayatsu et al., 2008). The effect of Po on *phoD*-harboring bacterial community richness was higher under phosphite than under phosphate fertilizer, possibly because AP from phosphate inhibited the transformation of Po into AP. Thus, phosphite-caused phosphate deficiency might promote the transformation of Po by microorganisms.

The SOC, TN, pH, and AP have been shown to play important roles in shaping the *phoD*-harboring bacterial community under phosphate conditions (Ragot et al., 2016; Wang et al., 2022), consistent with our results. Based on the SEM result, the effects of phosphate and phosphite fertilizers on soil properties and bacterial community in alfalfa fields were indeed different. The *phoD*-harboring bacterial diversity and richness were positively regulated by the pH under phosphite fertilizer treatment. However, the *phoD*-harboring bacterial diversity and richness act as negative and positive regulation to the ALP activity, respectively (Chen et al., 2019a; Wang et al., 2022). Wang et al. (2022) reported a similar SEM under phosphate fertilizer. However, the SEM was different between phosphate-treated and phosphite-treated soil, suggesting that Pi increased ALP activity while AP increased *phoD*-harboring bacterial richness under phosphite fertilizer. This might be partly because microbes could not consume the phosphite-produced Pi and AP. Thus more available phosphate transformed by ALP activity or indirectly by *phoD*-harboring bacterial richness was required in local soil ecosystem. It is speculated that the *phoD*-harboring bacterial community was altered by phosphite by altering the pH. To our knowledge, this is the first study to report the changes in soil properties and *phoD*-harboring bacterial community in response to phosphite treatment in the alfalfa field and could provide information for the phosphite utilization in the future. Although several articles have reported the changes in microbial communities caused by short-term habitat changes (Lazcano et al., 2012; Xiong et al., 2014), long-term phosphite fertilizer may also impact soil and microorganisms, which should be further explored. Since this study only evaluated the effects of phosphorus, ALP, and *phoD*-harboring bacterial community on alfalfa fields under short-term phosphite application, future studies should conduct a comprehensive diversity analysis of *phoA*- and *phoX*-harboring bacterial community. This will provide a further understanding of the phosphite effects and the bacterial responses to phosphates.

## Conclusion

This study evaluated the changes in soil properties and *phoD*-harboring bacterial community caused by phosphate and phosphite. Both phosphate and phosphite fertilizers increased soil TP and AP contents; however, the phosphite-induced increase of TP and AP was lower than that caused by phosphate. TP, Pi, and AP negatively regulated the ALP activity, and the *phoD*-harboring bacteria diversity was regulated by phosphate availability in the soil. The diversity of the *phoD*-harboring bacterial community was higher in the phosphite-treated soil compared to phosphate-treated soils. Furthermore, Proteobacteria, Firmicutes, and Acidobacteria phyla were identified. Frankiaceae, Sphingomonadaceae, Rhizobiaceae were significantly correlated with ALP activity in phosphite-treated soil. The results demonstrated that phosphate fertilizer significantly affected the ALP activity by regulating *phoD*-harboring bacterial diversity and richness through altered SOC, TN, pH, and AP. The pH positively regulated the *phoD*-harboring bacterial community under phosphite compared to phosphate fertilizer treatment. Moreover, the different responses of the *phoD*-harboring bacterial community to phosphate and phosphite fertilizer demonstrated the difference in ALP activity in the alfalfa field.

## Data availability statement

The raw data supporting the conclusions of this article will be made available by the authors, without undue reservation. The data presented in the study are deposited in the NCBI Sequence Read Archive (SRA) database, accession number: PRJNA874476.

## Author contributions

ZL: conceptualization, investigation, methodology, formal analysis, original draft, writing and review, and project administration. JW: investigation, methodology, analysis, and original draft. JH: investigation and formal analysis. YW: investigation, formal analysis, and revise. LC: formal analysis and revise. CY: investigation, methodology, and formal analysis. JF: review, editing, and supervision. JS: project administration, conceptualization, and supervision. All authors contributed to the article and approved the submitted version.

## Funding

This work was supported by Natural Science Foundation of Shandong Province (No. ZR2020QC185), China Forage and Grass Research System (CARS-34), Doctoral Scientific Research Startup of Qingdao Agricultural University (No. 6631119038).

## Conflict of interest

The authors declare that the research was conducted in the absence of any commercial or financial relationships that could be construed as a potential conflict of interest.

## Publisher’s note

All claims expressed in this article are solely those of the authors and do not necessarily represent those of their affiliated organizations, or those of the publisher, the editors and the reviewers. Any product that may be evaluated in this article, or claim that may be made by its manufacturer, is not guaranteed or endorsed by the publisher.

## Footnotes

^1^ http://www.r-project.org
