# Supplementary material for: Changes in soil properties and the phoD-harboring bacteria of the alfalfa field in response to phosphite treatment
Source: Front Microbiol. 2022 Nov 29;13:1013896. doi: 10.3389/fmicb.2022.1013896 (PMC9746240; doi:10.3389/fmicb.2022.1013896)
Supplement: Supplementary file 1 [file Data_Sheet_1.zip › Supplementary Table 5.docx]

**Supplementary Table 5** The result of detrended correspondence analysis

|  | **DCA1** | **DCA2** | **DCA3** | **DCA4** |
| --- | --- | --- | --- | --- |
| Eigenvalues | 0.0496 | 0.0068 | 0.0140 | 0.0154 |
| Decorana values | 0.0500 | 0.0079 | 0.0029 | 0.0022 |
| Axis lengths | 0.7413 | 0.3457 | 0.3741 | 0.4080 |

Note: Detrended correspondence analysis with 26 segments. Rescaling of axes with 4 iterations.
