# Supplementary material for: Changes in soil properties and the phoD-harboring bacteria of the alfalfa field in response to phosphite treatment
Source: Front Microbiol. 2022 Nov 29;13:1013896. doi: 10.3389/fmicb.2022.1013896 (PMC9746240; doi:10.3389/fmicb.2022.1013896)
Supplement: Supplementary file 1 [file Data_Sheet_1.zip › Supplementary Tables 1 and 2.docx]

**Supplementary Table 1** The significant levels of the soil properties following the application of phosphate and phosphite fertilizers at different concentrations.

| **Index** | ***F* value** | ***P* value** |
| --- | --- | --- |
| TP | 18.394 | ＜0.001 |
| Po | 1.155 | 0.373 |
| Pi | 20.928 | ＜0.001 |
| AP | 19.509 | ＜0.001 |
| TN | 1.103 | 0.404 |
| AN | 0.393 | 0.925 |
| AK | 1.596 | 0.184 |
| SOC | 0.779 | 0.638 |
| TN/TP | 4.045 | 0.004 |
| SOC/TP | 5.765 | 0.001 |
| pH | 1.455 | 0.231 |
| ALP | 5.159 | 0.001 |
| ACP | 29.023 | ＜0.001 |

Note: Abbreviations are as followed: TP, total phosphorus (g kg^-1^); Po, organic phosphorus (g kg^-1^); Pi, inorganic P (g kg^-1^); AP, available phosphorus (mg kg^-1^); TN, total nitrogen (g kg^-1^); AN, alkaline dissolved nitrogen (mg kg^-1^); AK, available potassium (mg kg^-1^); SOC, soil organic carbon (g. kg^-1^); ALP, soil alkaline phosphatase (μmol d^-1^ g^-1^ soil); ACP, soil acid phosphatase (μmol d^-1^ g^-1^ soil); TN/TP, total nitrogen to total phosphorus ratio; SOC/TP, soil organic carbon to total phosphorus ratio.

**Supplementary Table 2** Soil properties under different concentrations of phosphate and phosphite fertilizer treated conditions

| **Treatment** | **Po** | **TN** | **AN** | **AK** | **SOC** | **ACP** | **TN/TP** | **SOC/TP** |
| --- | --- | --- | --- | --- | --- | --- | --- | --- |
| CK | 0.56±0.02a | 0.70±0.02a | 54.26±8.13a | 149.89±8.61b | 9.01±0.26a | 28.48±1.06c | 1.20±0.04a | 15.50±0.54a |
| CK-K | 0.52±0.01a | 0.68±0.05a | 50.83±0.72a | 178.46±8.43ab | 8.88±0.43a | 49.07±0.76a | 1.22±0.10a | 15.94±0.96a |
| Pi-30 | 0.52±0.01a | 0.66±0.01a | 60.15±11.50a | 170.03±8.28ab | 8.63±0.18a | 47.17±1.66a | 1.10±0.01a | 14.45±0.18a |
| Phi-30 | 0.52±0.04a | 0.69±0.01a | 48.60±5.07a | 202.20±22.12a | 8.78±0.10a | 45.53±0.93a | 1.23±0.04a | 15.68±0.22ab |
| Pi-60 | 0.58±0.02a | 0.70±0.03a | 49.38±3.36a | 173.18±14.51ab | 9.07±0.18a | 39.69±0.18b | 1.12±0.04a | 14.52±0.06ab |
| Phi-60 | 0.56±0.03a | 0.70±0.03a | 51.62±7.07a | 182.81±16.22ab | 8.71±0.40a | 45.85±0.57a | 1.19±0.05a | 14.82±0.71ab |
| Pi-90 | 0.58±0.03a | 0.74±0.02a | 55.22±4.67a | 161.67±6.11ab | 9.28±0.18a | 36.27±1.77b | 1.14±0.07a | 14.18±0.49ab |
| Phi-90 | 0.56±0.04a | 0.71±0.03a | 49.27±4.58a | 184.33±9.27ab | 8.76±0.22a | 48.01±0.48a | 1.17±0.09a | 14.45±0.89ab |
| Pi-120 | 0.58±0.02a | 0.67±0.00a | 48.60±1.43a | 200.87±14.45a | 8.81±0.08a | 45.25±2.37a | 0.85±0.03b | 11.29±0.40c |
| Phi-120 | 0.58±0.02a | 0.73±0.02a | 50.84±3.8a | 180.62±10.35ab | 9.23±0.24a | 38.11±0.29b | 1.06±0.03a | 13.39±0.31b |

Note: The abbreviations are same as Table S1. the other abbreviations are as followed: CK, non-fertilized control; CK-K, potassium fertilized control; Phi, phosphite fertilizer; Pi, phosphate fertilizer, Po, organic phosphorus; TN, total nitrogen; AN, alkaline dissolved nitrogen; AK, available potassium; SOC, soil organic carbon; ACP, acid phosphatase activity; TN/TP, total nitrogen to total phosphorus ratio; SOC/TP, soil organic carbon to total phosphorus ratio. The treatment values 30, 60, 90, and 120 indicate 30, 60, 90, and 120 mg P_2_O_5_ kg^-1^ soil, respectively. The data are shown as the means ± standard errors (n = 3). Different lowercase letters indicate the significant differences (*P* < 0.05) in each treatment.
