# Supplementary figures and images for: Changes in soil properties and the phoD-harboring bacteria of the alfalfa field in response to phosphite treatment
Source: Front Microbiol. 2022 Nov 29;13:1013896. doi: 10.3389/fmicb.2022.1013896 (PMC9746240; doi:10.3389/fmicb.2022.1013896)

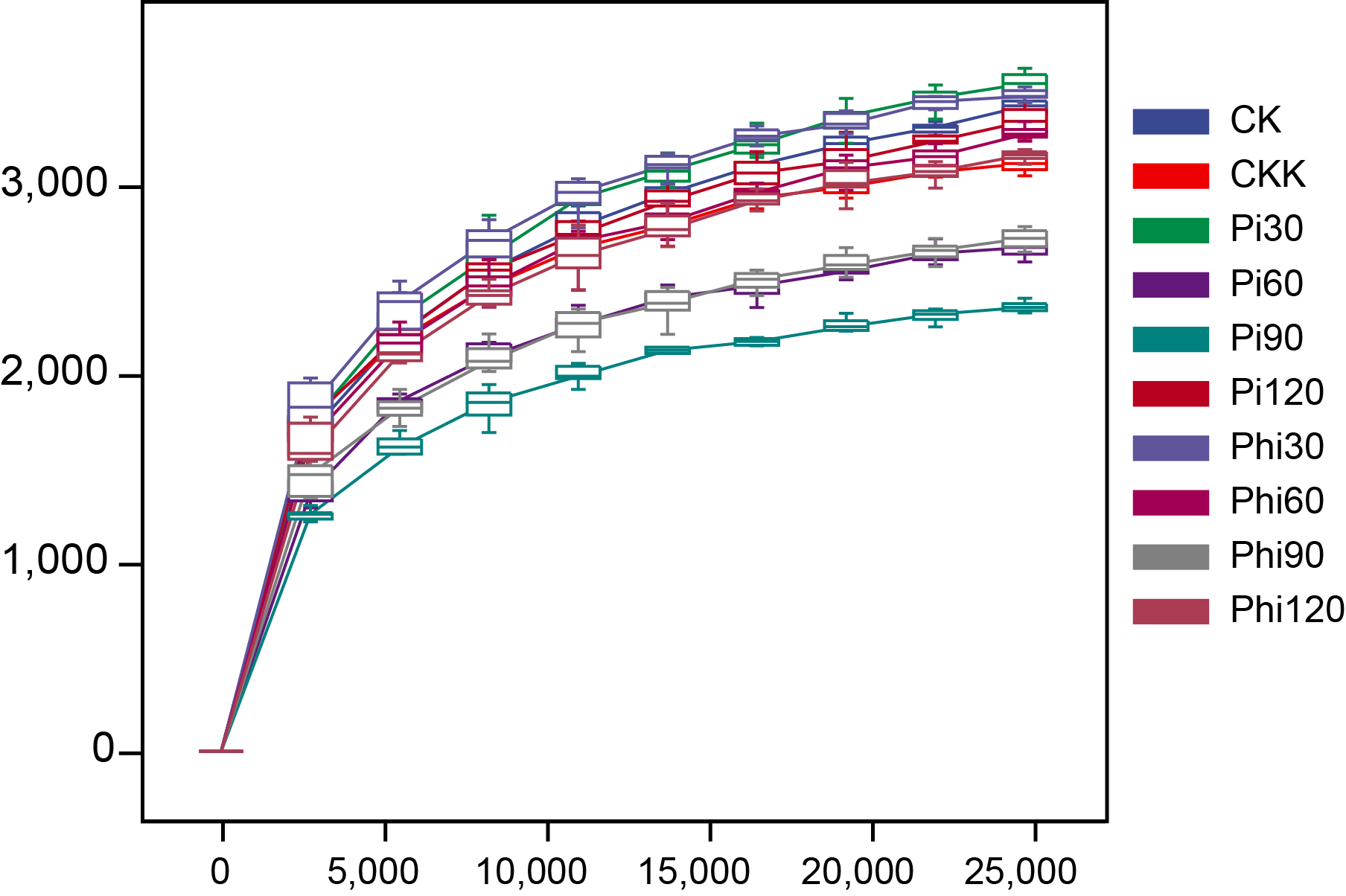

Supplement: Supplementary file 2 [file Image_1.JPEG]

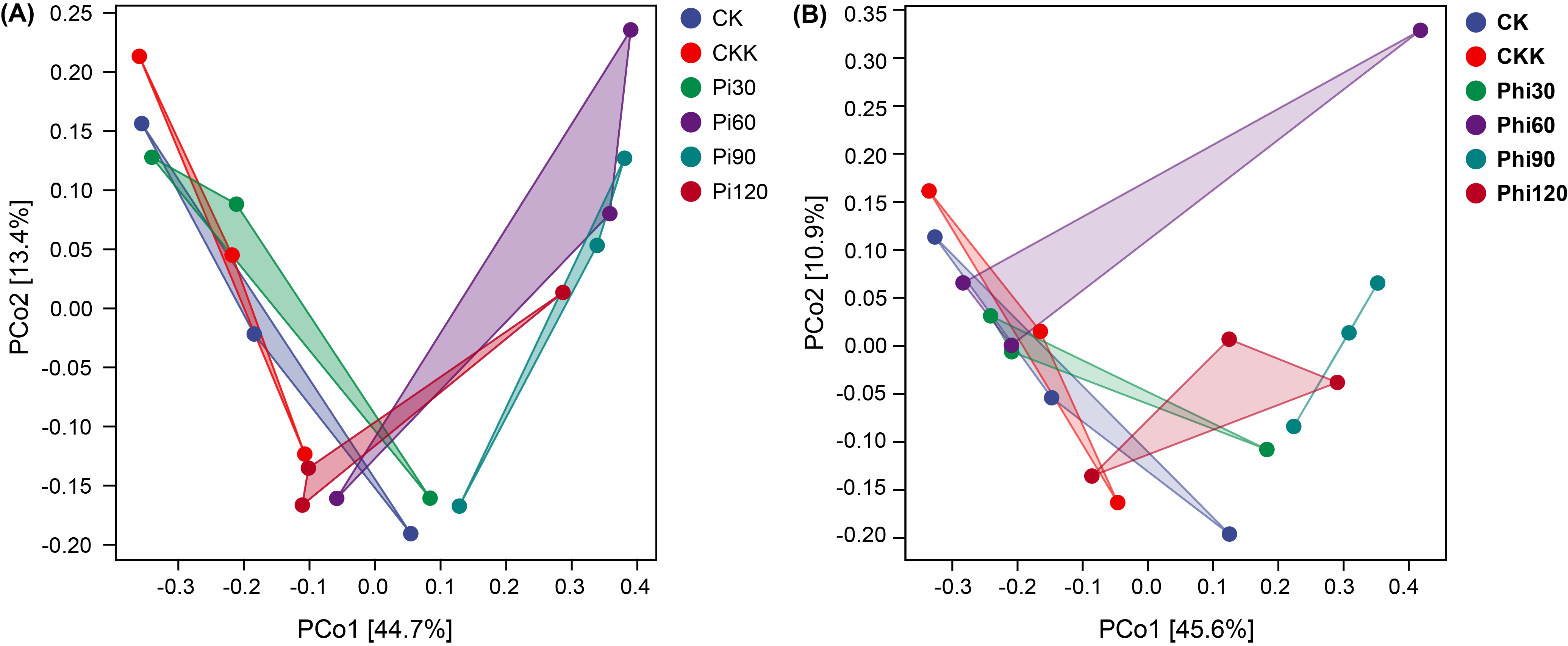

Supplement: Supplementary file 3 [file Image_2.JPEG]

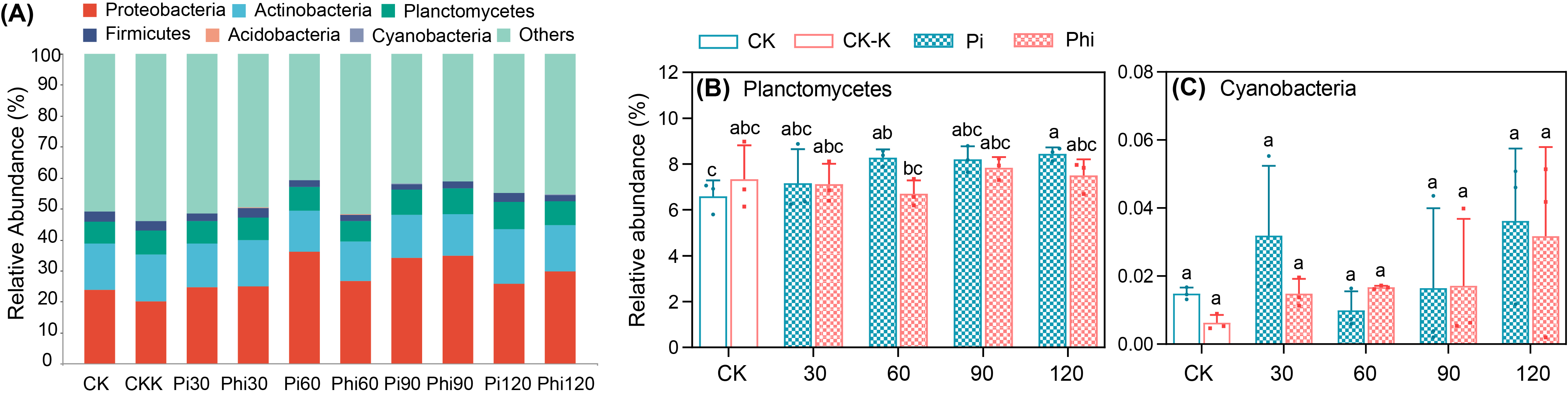

Supplement: Supplementary file 4 [file Image_3.JPEG]

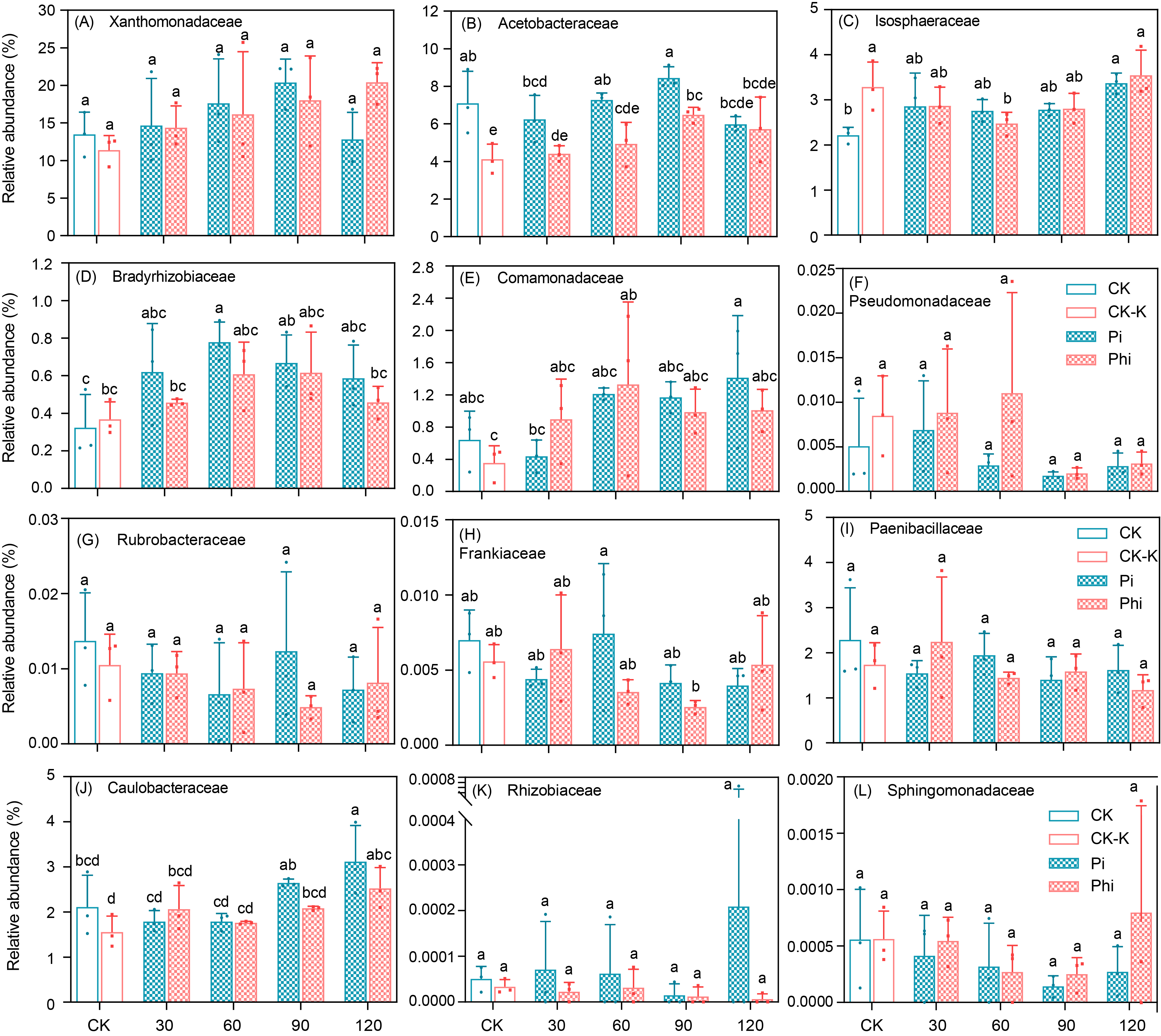

Supplement: Supplementary file 5 [file Image_4.JPEG]

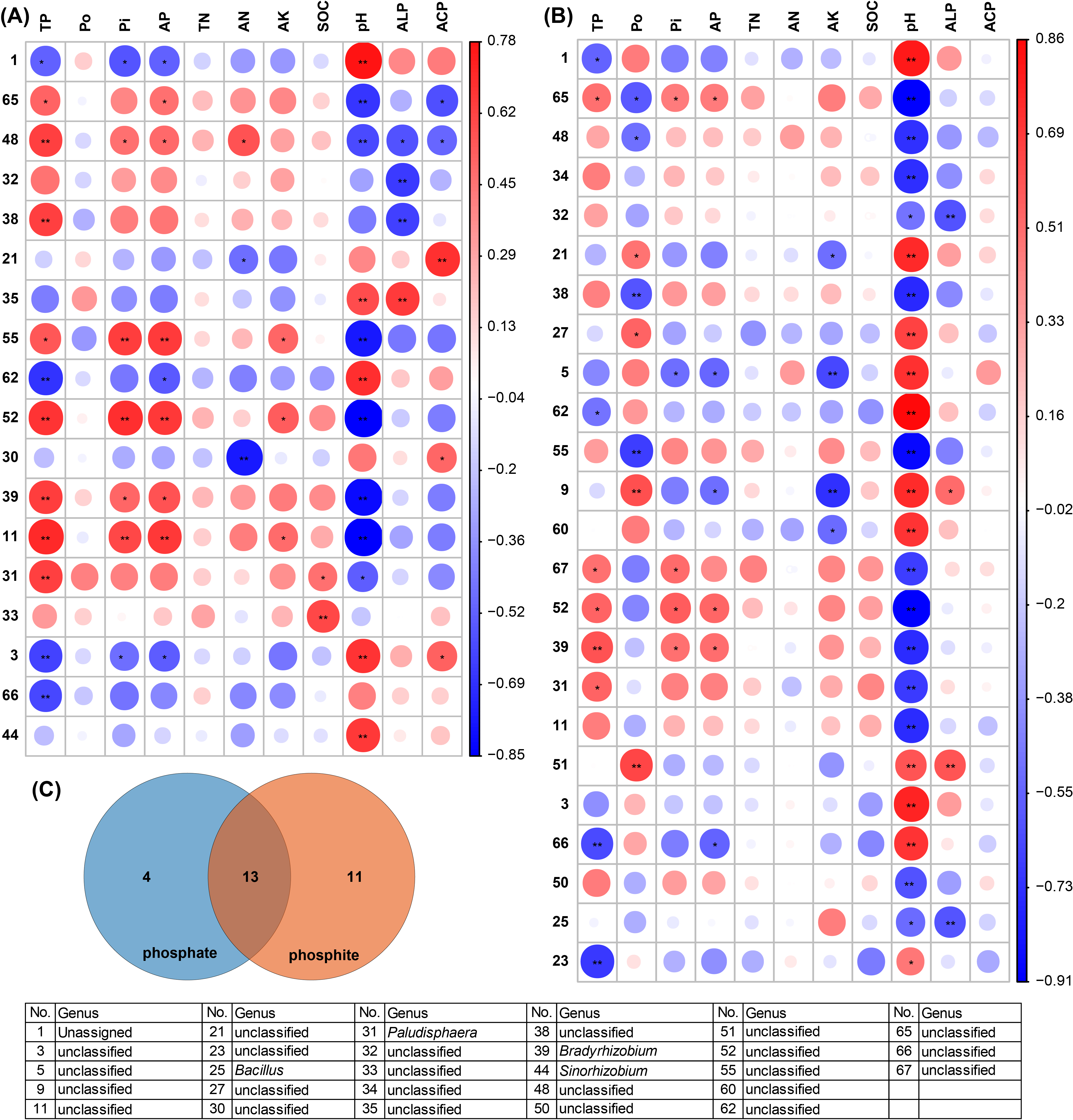

Supplement: Supplementary file 6 [file Image_5.JPEG]
